# Intensive Care Nurses’ Belief Systems Regarding the Health Economics: A Focused Ethnography

**DOI:** 10.5539/gjhs.v8n9p172

**Published:** 2016-01-22

**Authors:** Abbas Heydari, Ali Vafaee-Najar, Mahmoud Bakhshi

**Affiliations:** 1 Evidence-Based Care Research Center, School of Nursing and Midwifery, Mashhad University of Medical Sciences, Mashhad, Iran; 2 Health Sciences Research Center, School of Health, Mashhad University of Medical Sciences, Mashhad, Iran

**Keywords:** culture, ethnography, health care economics, Intensive Care Unit

## Abstract

**Background:**

Health care beliefs can have an effect on the efficiency and effectiveness of nursing practices. Nevertheless, how belief systems impact on the economic performance of intensive care unit (ICU) nurses is not known. This study aimed to explore the ICU nurses’ beliefs and their effect on nurse’s: practices and behavior patterns regarding the health economics.

**Methods:**

In this study, a focused ethnography method was used. Twenty-four informants from ICU nurses and other professional individuals were purposively selected and interviewed. As well, 400 hours of ethnographic observations were used for data collection. Data analysis was performed using the methods described by Miles and Huberman (1994).

**Findings:**

Eight beliefs were found that gave meaning to ICU nurse’s practices regarding the health economics. 1. The registration of medications and supplies disrupt the nursing care; 2. Monitoring and auditing improve consumption; 3. There is a fear of possible shortage in the future; 4. Supply and replacement of equipment is difficult; 5. Higher prices lead to more accurate consumption; 6. The quality of care precedes the costs; 7. Clinical Guidelines are abundant but useful; and 8. Patient economy has priority over hospital economy. Maintaining the quality of patient care with least attention to hospital costs was the main focus of the beliefs formed up in the ICU regarding the health economics.

**Conclusions:**

ICU nurses’ belief systems have significantly shaped in relation to providing a high-quality care. Although high quality of care can lead to a rise in the effectiveness of nursing care, cost control perspective should also be considered in planning for improve the quality of care. Therefore, it is necessary to involve the ICU nurses in decision-making about unit cost management. They must become familiar with the principles of heath care economics and productivity by applying an effective cost management program. It may be optimal to implement the reforms in various aspects, such as the hospital’s strategic plan and supply chain management system.

## 1. Introduction

Scarcity of resources is a fact in all health care systems. This issue is especially explicit when caring for critically ill ICU patients, because these patients are in need of the resource-intensive technologies, special medicines, and highly specialized personals (Cox, Laupland, & Manns, 2006). ICUs consume a large proportion of hospital resources and are focused places in the hospital with well-defined boundaries (Cantlupe, 2011; Kahn, 2006). Some studies show that a considerable proportion of resources are spend on futile care in the ICUs that as well as costs, would increase the complications such as bedsores, catheter-related infections and ventilator-associated pneumonia (Neuberg, 2009; Niderman & Berger, 2010). Therefore, health economics may play an important role in guiding decision-making in the intensive care units (ICUs) (Cox et al., 2006).

Health economics is a branch of economics that studies and evaluates the quantity, quality, and value of the limited resources available to healthcare systems, as well as determining how these factors best combine to produce certain services, with the aim of maximizing effectiveness and efficiency (Martinez-Giralt & Barros, 2013). Nurses as the largest professional group of health care providers and the main users of resources (Ntlabezo, Ehlers, & Booyens, 2004) can make rational use of resources, waste control, and improve the patients’ outcomes in order to play an important role in the economic management of nursing services (Oliveira, Rodrigues, Haddad, Vannuch & Taldivo, 2012). Shortage of resources, administrative practices, and organizational culture are among the factors that impact on nursing care and use of resources (Holland, 1993). As well, the ICU environment with certain characteristics such as critically ill patients, high mortality rate, unpredictable nature of the work, high use of resources, and more expensive equipment have led to a specific care culture (Guidet & González-Romá, 2011; Scott, Estabrooks, Allen, & Pollock, 2008). Within this context, culture and beliefs can have an effect on the efficiency of nursing activities and resource consumption method (Scott et al., 2008).

This study was based on the work of Checkland (2007) and Weick (2001). They argue that beliefs of peoples about their roles are influenced by which how they make sense of their role responsibilities. This sense making was informed by previous experiences and underlying values. The sense making will impact on which items they notice in the world around them; this in turn will determine how they act in practice (Checkland, 2007). Therefore, basic beliefs that the nurses hold regarding the sense making of their role will influence how they deliver care to patients, how they audit that care, and how they react to any resource deficit (Weick, 2001).

The identification of ICU nurses’ beliefs about health economics can help to better understand their clinical practice and make better decisions for cost management and quality of care in the ICU (Witter, Ensor, Jowett, & Thompson, 2014). Although several studies have been conducted on the perspective and knowledge of nurse managers and staff nurses on health economics (Heydari, Mazloom, Vafaee-Najar, & Bakhshi, 2015), and the impact of organizational culture and working environment on nursing care (Margaret Fry, 2012; Manojlovich & Laschinger, 2008; Squires & Juárez, 2012), the impact of nurses’ belief systems on economic performance is unknown. Oliveira et al. showed that nurses don’t attention to the costs of their clinical activities because they focus on care quality issues (Oliveira et al., 2012). Similar findings were also reported by another studies in which quality practice was important to the nurses practicing in the ICU (Storesund, & McMurray, 2009; Williams, 1998).

In Iran, public hospitals have a high operating costs and low efficiency, despite the large amount of resources that are allocated to them. This aroused questions regarding how the use of resources by the public hospitals (Aboulhallaje, Hatamabad, & Abachizadeh, 2011). In a qualitative study, participants believed that 80% of ICU care is futile (Yekefallah, Ashktorab, Manoochehri, & Hamid, 2015). Given the high costs of ICU care, it may be necessary to investigate the beliefs of Iranian ICU nurses with regard to health economics. Thus, considering the key role of cultural beliefs ICU nurses on their performance (Durbin, 2006), this study aimed to explore and understand the ICU nurses’ beliefs and how it effect on their practices and behavior patterns regarding the health economics. In such circumstances, nurse managers and staff nurses can criticize their own belief systems with more awareness and provide appropriate changes for delivery a more reasonable and cost-effective care services.

## 2. Methods

The focused ethnography method was used in this study. This method helps better understand the nurses’ experiences of daily work, context, and processes in the clinical units (M. Fry & Stainton, 2005). Focused ethnography, also allow researchers to focus on specific topics instead of the whole and it is used when the researchers are closely familiar with the field of study (Knoblauch, 2005). In this study, the first author who was familiar with cultural norms of the research setting, due to the long employment in the ICUs as a clinical nurse as well as a clinical instructor for nursing students in medical and surgical ICUs.

In Iran, Public hospitals are funded mainly through the financial resources of the government that are allocated to hospitals by the Ministry of Health and Medical Education. The salaries and other employee benefits are paid by government funds based on a fixed pay rate (Moosaniaye-Zare, Asefzadeh, alijanzadeh, & Sanei, 2014). The study setting was 4 ICUs in two teaching public hospitals affiliated to Mashhad University of Medical Sciences, Mashhad, Iran (one medical and one surgical ICU in each hospital). The hospitals were the largest centers among the referral centers and located in Mashhad, Iran. The mission of these hospitals was to provide the most favorable care for patients in the country with the aim of increasing the satisfaction and trust of patients through continuous quality improvement. In this setting, the ICUs bed occupancy rate ranged between 95% and 100%. The number of beds in the different ICUs ranged from 8 to 15 beds per adult unit and the number of nurses varied from 18 to 35 individuals. The supply and distribution system of materials and equipment were the same and focused for all units. So that, the hospital supply center was responsible for providing medicines and the supplies and equipment required for entire units of the hospital. The supplies were stored in the central pharmacy and warehouse after purchase. The ICUs submitted their requests for medication list as well as supplies and equipment they needed via Hospital Information System (HIS).

### 2.1 Data Collection

Data collection had two components; (a) observation of unit activities and existing behavior patterns, (b) formal and informal interviews with unit nurses and other health professionals. The purpose of the data collection was to obtain data from a variety of sources and perspectives in order to provide a complete understanding of the beliefs and behavioral patterns of the participants regarding the health economics.

Observations were done as a participant observer while engaging in the clinical education of nursing students in ICUs. The observations were conducted during all weekdays except holidays within all four ICUs under study, from October 2013 to May 2014 for 9 months. The observations were made first in a descriptive method and as the study progressed, they were conducted in selective and focused forms, respectively (Spradley, 1980). During the observations, the patient care activities; unit based managerial activities; how to order, deliver, store and use the supplies and equipment, as well as the documents of the unit and hospital were recorded as field notes. In addition, the researcher recorded the casual conversations and quoted stories by participating in the meetings, conferences, and break room.

Interviews were done informally during the observations and formally through semi-structured interviews. Two main questions in the interviews included: (a) how do you explain your experience of nursing care efficiency at the workplace? (b) how do you describe the use of supply and equipment during daily work routin e? Also, some probing questions were asked to seek more information and clarify the responses.

The interview durations ranged between 15 minutes to 2 hours and they were recorded using a recorder. Interviews were done in a quiet environment at the participants’ workplace. All the interviews were conducted by the first author. Formal interviews were conducted with 24 key informants. Table 1 shows the characteristics of the 24 interviewed participants.

**Table 1:** Informants’ Characteristic of the study

Age (M±SD)		39±4.32

Work Experience (M±SD)		13±4.6

Gender	Male (n)	8
	Female (n)	16

Job position	Head Nurse (n)	4
	Assistant Nurse (n)	4
	Clinical Nurse (n)	8
	support staff (n)	3
	Supervisor (n)	3
	Physicians (n)	2

Key informants were selected through maximum variation sampling among the head nurses, assistant head nurses, nurses responsible for equipment and stock from all four ICUs. The inclusion criteria were having a minimum of two-year work experience in clinical nursing and holding Bachelor’s or Master’s degrees in nursing. According to ethnographic sampling, good informants are ones that have enough experience on the phenomenon studied, are confident within their field and willing to discuss their experiences (Morse, 2007; Wolcott, 2008). In addition to validating the finding, interviews were performed with other health professionals (physicians, supervisors, nursing aids, and secretaries).

### 2.2 Ethical Considerations

The study design was approved by the ethical committee of Mashhad University of Medical Sciences. Before each interview, all participants were informed about the goals and methods of the study. As well, they were informed that participation in this study is voluntary and they could be excluded any time they desired. Informed consent forms were filled out by all those interviewed. Participants were assured that their responses will be remained confidential.

### 2.3 Data Analysis

Data management and analysis was performed simultaneously with data collection using the methods described by Miles and Huberman (Miles, Huberman, & Saldana, 2013). Data collection continued until saturation and a rich description of the phenomenon under study was achieved. Following each field work, data were converted into write up. Then, using the contact summary sheet was prepared a general summary of the main points of the field. In addition to providing a general understanding of the data, this sheet is a good guide to plan next contacts (Miles et al., 2013). The recorded ethnographic interviews were transcribed.

Data analysis was conducted through a process of analytical circling and with an inductive reasoning approach. Analysis processes are broadly aligned to spradley’s (1980) three phases of fieldwork: descriptive, selective and focused. In descriptive phase of fieldwork, researcher initially read the data to obtain a general understanding of them. Then, the texts of the data were examined in terms of their meanings and were coded in order to identify the descriptive codes or concepts. After conducting this procedure for several participants, a list of all descriptive concepts was collected. Descriptive concepts were summarized and classified into a smaller number of sets through the use of map making. Analysis then progressed until 19 key events or cultural themes were extracted to identify beliefs and behaviors of nurses regarding the health economics (Miles et al., 2013). The selective phase of fieldwork involved a more in-depth study in relation to 19 themes elicited from the descriptive phase of fieldwork. As fieldwork progressed pattern coding was utilized, where descriptive concepts and cultural themes were grouped and collapsed into 9 themes. The focused phase of fieldwork was spent validating 9 cultural themes and undertaking ethnographic interviews. Finding from interviews were compared with findings from observations. The final themes were used to develop causal network that linked observed behaviors with expressed beliefs by participants (Miles et al., 2013).

### 2.4 Trustworthiness

Trustworthiness was maintained by using strategies of credibility, confirmability, dependability and transferability as described by Lincoln and Guba (1985). To this end, the following strategies were used: (a) extensive sampling of interview participants; (b) triangulation in data collection; (c) purposeful sampling of unit events for observation; (d) prolonged engagement in field observations; (e) member checking; and (f) completion of an audit trail that documented all results and extracted interpretations from the data. During the analysis, the interpretations were discussed in details by research team through regular sessions. The final analysis was reviewed by team members as well as by other researcher who was familiar with the research methodology.

## 3. Findings

The study population was composed of 106 nurses who performed patient care activities at 4 ICUs at two large public hospitals in Mashhad, Iran. The total observation time was about 400 hours. Nurses aged 25 to 48 years (M = 34, SD = 7.17) and they all had a bachelor’s degree in nursing and a working experience of 2 to 26 years (M = 9, SD = 8.6). ICU nursing staffs include a head nurse (unit nurse manager), one assistant head nurse, and several clinical nurses in each working shift. The regulatory and administrative tasks were mostly performed by the head nurse and assistant head nurse, and each clinical nurse was responsible to take care of two patients.

Observations and interviews of ICU nurses identified a knowledge system that was made up of eight beliefs that helped regulate and sustain an understanding of nursing practices regarding the data registration; maintenance and use of supplies and equipment; quality of care; and patient and hospital costs (Table 2).

**Table 2:** Eight embedded beliefs within critical care practices regarding the health economics

*The registration of medications and supplies disrupt the nursing care Monitoring and auditing improve consumption*
*There is a fear of possible shortage in the future*
*Supply and replacement of equipment is difficult*
*Higher prices lead to more accurate consumption*
*The quality of the care precedes costs*
*Clinical Guidelines are abundant but useful*
*Patient economy has priority over hospital economy*

***The registration of medications and supplies disrupt the nursing care***

This idea arises from the fact that nurses should record all consumed items in the hospital information system (HIS). In the binder of the unit, a guideline was issued by hospital matron to nurses in which the significance of accurate record of consumed items even cotton, iodine and alcohol were emphasized.

The nurses believed that registration of medicines and supplies in the HIS reduces the quality of nursing care because it is too simple task for nurses and even time consuming. One nurse Said:

*Registration the consumed items take so much time. It causes us to lose time to take care of our patients, while most ICU patients are critically ill and require constant care (ICU D)*.

Interviews with nurses showed that there was a negative attitude towards this task. One nurse stated:

*I hate the registration of medicines and consumables. This is not part of our duty. This is not a specialist’s task. A secretary can do it, too (ICU C)*.

Field notes showed that nurses sometimes did not register the used medications and supplies in the HIS accurately. In some cases; angiocath, catheter, and serum were recorded less than the actual amount of consumption.

***Monitoring and auditing improve consumption***

The observations and interviews with informants indicated that the consumption of supplies and medications in the ICU is monitored by hospital managers. This supervision is mainly provided in a periodic form by central pharmacy, warehouse and accounting department, and as well constantly by ICU head nurse and assistant head nurse.

Nurses believed that the audit and control of resource consumption in the unit increases the efficiency. One nurse stated:

*Along with more monitoring, medications and supplies are used more accurately with precise accounting and control. Control and strictness by the management system has led to savings in the consumption of the items (ICUA)*.

The recorded used items were compared in the patient’s chart and the HIS for controlling the medicine and supplies use. In cases where the consumption in the unit was not matched with the HIS records, the nurses in charge were questioned and they had to make it clear.

***There is a fear of possible shortage in the future***

ICU inventory was particularly important for ICU nurses. Due to the fear of a possible shortage of some items, head nurses and nursing staffs try as much as possible to maximize the inventory, and they store the supplies. In this regard, one head nurse expressed:

*Sometimes I fear that a device does not exist in the unit, therefore I try to increase my inventory in order to tackle the shortcomings. One of the reasons that sometimes some of items are available in the unit excessively is to be prepared for the circumstances in which there might be a shortage (ICU A)*.

Field notes confirmed occasional shortage of consumables in the unit, so that the lack of items such as ventilator set, normal saline serum, and some medications were recorded on different days.

In case of shortage, nursing staffs provide the needed medicine or supplies from the other units of the hospital or to be achieved by the patient’s family from the outside of hospital. This condition increased workload and workplace stress on nurses and can make a delay in delivery of patient care. A nurse assistant said:

*We have to bear a lot stress, when we don’t have medicines, when we don’t have the supplies we need. If we have to ask a family member to provide something, he/she gets upset, but the fact is that shortage of supplies is not the nurse’ problem (ICU C)*.

***Supply and replacement of equipment is difficult***

Due to limited financial resources, purchasing expensive equipment for hospital was a long process. This encountered ICU head nurses face serious problems with the provision and replacement for expensive equipment in the unit and put more emphasis on the maintenance and use of their equipment. One head nurse stated:

*We feel annoyed the time when we want to ask for some devices from our system. To buy a mattress, we lose lots of energy. The replacement of devices and equipment is really time-consuming and difficult (ICU D)*.

Sometimes, other factors such as fear of reprimands makes the head nurses monitor the proper use of equipment. One head nurse mentioned:

*It is a difficult process to get and replace tools and equipment; in addition, I must be responsive to the manager about the equipment failure (ICU A)*.

The assistant head nurse monitors the performance of equipment and advice seriously at the beginning of each shift. As well, one of the clinical nurses was selected as an agent and monitored the function of the equipment in the unit. Along with most of the equipment such as monitors, ventilators, and the ECT were also installed a training manual for their proper use. The hospital management has also issued a checklist to each unit in order to evaluate the performance of various pieces of equipment.

Nursing staffs were skilled enough to use the equipment accurately and safely. A specific place was intended for the maintenance of equipment in the unit. Some equipment such as ventilators, air mattresses, and monitoring devices were used for a long time at the ICUs. One head nurse said:

*I have delivered four air mattresses about 18 years ago. I stressed the nurses needed to be careful and make sure that the needle does not damage to the mattress… These mattresses are still being used. A few days ago, I contact with support firm mattress for service them; they were surprised that we still use these mattresses (ICU C)*.

***Higher prices lead to more accurate consumption***

The price of equipment and supplies was one of the factors affecting their consumption management. Cheap and available medicines and supplies were not carefully used. The field notes showed that items such as cotton, iodine, suction catheter which were abundant in the unit stock, had more wastes. In contrast, expensive items were used more accurately and carefully. Some certain medications were given to the ICU nurses in the form of “just-in-time” for reasons such as high cost, low consumption, and scarcity. A head nurse assistant stated:

*We have lots of catheters within the unit because they are not expensive. The hospital warehouse delivers them as many as we ask for. But things like double lumen and CVP catheter should be received with care and that depends on their prices (ICU B)*.

The prices of supplies and equipment had an impact on the quality of care. It takes extra care to prevent complications for expensive items. One nurse said:

*Things like ventriculostomy and CVP catheters are high-priced, so the nurses know they should be careful not to take it out at the time of patients’ change position, because if it is out; not only the patient will suffer side effects, but also there will be about three hundred dollars loss (ICU A)*.

***The quality of the care precedes costs***

Nurses mainly focused on the quality of their caring activities rather than their costs. A nurse stated:

*I don’t mind hospital expenditure so much. I take care of the patient. My work and its quality are of utmost importance (ICU C)*.

A strong motivational factor for the nurses to prevent infection and to faster hospital discharge was founded on the belief in providing high-quality care for patients. One nurse said:

*A good nurse is someone with a good-quality performance and moves the patient faster towards earlier discharge from the ICU. Being hospitalized in the ICU involves numerous side effects (ICU B)*.

Some infection prevention measures are taken such as periodic sampling of different parts of the unit, the use of special glasses and masks, as well as holding educational classes. One head nurse assistant stated:

*Prevention of infection is very important to us and we continuously caution the co-workers about washing hands. One in each unit is selected as infection controller and attends in hospital infection control meetings and transfers new contents to other personnel (ICU A)*.

Nurses preferred new costly instruments and technologies in their caring activities mainly because they lowered the side effects and eased their jobs. One nurse said:

*Currently, we use ready bandages in all cases because they have fewer side effects. We already cut the gas with scissors for dressing the tracheotomy. The threads entered the stoma and led to lots of problems for the patient (ICU D)*.

***Clinical Guidelines are abundant but useful***

One of the strategies employed to improve the quality of health care in the hospitals in this study was the clinical governance plan. In this plan, there was special emphasis on the implementation of caring standards or guidelines, and the nursing personnel were required to fulfill them.

Nurses believed that the caring standards they are obliged to do are abundant, but at the same time they considered the outcomes of their implementation favorable. Considering the benefits of implementing the caring standards, one head nurse assistant stated:

*Caring guidelines have led to specified and uniformed routes; so that everyone cannot follow their tastes. These guidelines improve the outcomes of activities, the patient is discharged earlier and there are fewer side effects (ICU B)*.

In this line, another nurse said:

*We may feel a lot of pressure at a point of time, but caring guidelines in the long term upgrade the caring activities, decrease bed sores, and lessen medication errors as well as death rates (ICU D)*.

***Patient economy has priority over hospital economy***

Field notes and documents of patients showed that the majority of ICU patients are not in a good economic situation. The nursing staff believed that ICU costs are high and the patients and their family bear heavy economic pressures. During the caring activities, the patient’s costs were more emphasized than hospital costs. Many of the nurses believed that because of the high costs of treatment, public hospitals should provide their patients with cheap or even free services. One nurse said:

*In principle, treatment should be free, something that is really mentioned in the laws. But in practice, it is not free. By the way, here is a training hospital so they can come into terms with the patient”*. Another nurse stated: *“When the issue of economy turns out, first of all I think about the patients because they are in bad physical conditions and cannot afford the costs (ICU A)*.

The observations and interviews with the nurses demonstrated that no training course and workshop on health economics were held for the nurses. In terms of the basic concepts of health economics, the nurses were not aware of cost savings and cost-effective methods. At the same time, the nurses did their best not to increase the patients’ costs as far as possible. This effort was greater especially for patients who were not in a good economic situation. One nurse said:

*We register the consumed items for each patient in their names as much as possible. Sometimes, we register fewer items for the patients who are in a weak economic situation (ICU B)*.

## 4. Discussion

The results of present study showed that a special culture of ICU care has formed up in the field of health economics. The main finding of this study indicated that the belief systems were focused significantly on quality of care. In other words, ICU nurses make sense of their role as “provider of high quality care”. This sense making has influenced on their beliefs in relation to health economics. The Eight beliefs have led to development of shared knowledge between the ICU nurses that gave meaning to their practices and behaviors. ICU nurses put all their efforts to maintain the quality of patient care at a desirable level through an increase in available resources (storage), even if there is the scarcity of resources in their units.

These belief systems from the perspective of health economics can be studied in two interrelated areas: (1) care delivery (produce) management, (2) resource consumption management.

In the area of care delivery management, the ICU nurses made efforts to provide appropriate and high-quality care to patients via implementing clinical guidelines and reducing patients’ costs. Many nurses gave priority to patient’s costs and overlooked the costs of hospital. These findings are consistent with the results of other studies (McKenna, Keeney, & Hasson, 2009; Oliveira et al., 2012; Storesund & McMurray, 2009). Oliveira et al. suggested that nursing managers must gain a different perspective on managing costs, and clinical nurses need to experience a cultural change in order to increase the efficiency of nursing care (Oliveira et al., 2012).

The last two decades have seen a worldwide focus on quality of care in health care and quality improvement programs and safe care are widely considered (Storesund, & McMurray, 2009). Although high quality care can largely increase the effectiveness of nursing care, cost control perspective should also be considered at targeting nursing care services (Warburton, 2009). If the nurses carried out activities such as care of futile patients, irrelevant duties, and secretarial work, in addition to the waste of resources, they would lose the time that they could be used more effectively for other patients (Yekefallah et al., 2015). This means that inefficient nursing care imposes the unnecessary financial and non-financial costs on health system; and the quality of care encounter challenges in situations where there is shortage of resources (Bentley, Effros, Palar, & Keeler, 2008; Warburton, 2009). Thus, it is necessary to consider costs when making clinical and managerial decisions in critical care.

The ICU culture has a direct relationship with its effectiveness and quality of care (Durbin, 2006). Nurse Managers (NMs) can either shape or maintain nursing unit cultures that positively or negatively impact patient outcomes. NMs must take proper strategies in relation to limited resources, motivating nursing staff, and elevating staff performance. These strategies must result in high quality and cost-effective patient care (Casida, 2007). The result of this study also showed that head nurses and assistant head nurses constantly monitored the rational use of medicines and supplies and emphasized on the proper use of the equipment. The monitoring was done mainly due to local concerns about the financial resource constraints and resource-related difficulties encountered by ICU nurses. In this context, unit costs and health care cost-effectiveness usually not considered. Since the NMs’ role is the implementation of the hospital’s strategic plan for patient care in the nursing units, NMs must take action in alignment his or her nursing unit culture with the hospital’s strategic goals and objectives (Casida, 2007). In the study context, the hospital’s mission was to provide the most favorable care for patients with the aim of increasing the satisfaction and trust of patients, and the ICU culture was shaped in this regard. Therefore, it seems that some reforms must be made in the hospitals’ strategic plans. If hospital managers consider the cost-effectiveness perspective in the strategic plans, NMs were made to align with suggesting changes to increase efficiency of care. In this case, the ICU nurses give more attention to economic issues such as the length of stay in the intensive care unit, and effective use of limited resources (Casida, 2007).

In this study, the participants were not aware about health economics and how to increase the effectiveness of nursing care. But they made any attempt to reduce costs, especially patient costs due consideration given to ethical and humanistic issues. However, given the growing costs of health services and the limited resources available, nurses’ awareness of, and participation in, logical justification of hospital costs, and suitable use of resources is of vital importance (Oliveira et al., 2012). Therefore, nurses are required to learn more about health economics and cost-effectiveness to gain better evidence on the basis of their professional context to improve the quality of care and care-related expenses (Warburton, 2009). In the study by Ntlabezo (Ntlabezo et al., 2004), most participants (92.3%) believed that to contain costs, should be held continuing educational classes for the nurses.

In field of resource consumption management, ICU nurses have a sense of futurism in the supply and use of resources. They were driven the consumption by taking the rules and guidelines as well as the available resources and their values into account. The ICU inventory was important for the head nurse and nursing staff due to fear of a likely shortage in the unpredictable context of the ICU; therefore, they try to increase their available inventory as much as possible and store the supplies and equipments.

Some other studies have shown that it may be tendencies to store excessively due to the ambiguity and uncertainty in supply of ICU resource (Davis, & Doyle, 2011; Kaur & Hall, 2001). There are various methods to order for supplies in a predictable way to keep an adequate stock (Landry, & Beaulieu, 2013). As well, designing and implementing a systematic supply chain strategy in hospitals can access high-quality supplies in the quantities and sizes need for ICU patients (Davis, & Doyle, 2011).

Monitoring and auditing the use of resources in the unit is one of the main methods to control costs. In this study, the nurses believed that supervision had a positive effect on resource management in the unit. Carruth and Carruth (2007) found that financial managers and nurse executives considered accounting systems were moderately or very effective for cost containment and believed that the precise monitoring of the use of supplies and equipment effectively decreases the costs (Carruth, & Carruth, 2007).

The ICU personnel play an essential role in routine care of instruments and equipment, especially cleaning, checking for damage and reporting any defects (Kaur, & Hall, 2001). This study revealed that there are beliefs such as high-priced equipment, their problematic supply, and the prevention of disorder in caring services which make the ICU nurses obliged to protect the equipment and monitor their performance. Ntlabezo *et al*. showed that nursing managers have a positive perception about the maintenance of equipment items and they believe that the equipment should be controlled periodically by people who are experts in this field (Ntlabezo et al., 2004).

According to the results, ICU nurses did not regard the registration of medicines and supplies in the HIS because it loses the time and reduces the quality of nursing care. In different studies, a variety of reactions and sometimes contradictory responses from nurses towards electronic registration of health information and consumables have been reported (Carayon et al., 2011; Laramee, Bosek, Shaner-McRae, & Powers-Phaneuf, 2010). Yekefallah et al. (2015) showed that ICU nurses believed that some activities such as registering drugs into the computer system is a futile activity and reduces productivity and leads to low-quality care (Yekefallah et al., 2015). Therefore, it is of importance to examine the existing challenges and problems in this field in order to create favorable changes in the implementation of the HIS and careful registration of medicines and materials (Carayon et al., 2011). Given the high workload of ICU nurses, it seems logical to entrust irrelevant duties to the executive secretary. This may increases the quality of care for critically ill patients in the ICU.

### 4.1 Limitation

Although we made efforts to maximize the diversity in the samples and the existing ICUs, the sample used in this study cannot be the representative of ICU culture regarding the resource management in all the ICUs in Iran. Therefore, the implications of the findings may be limited. However, the same care settings in Iran or abroad could make use of the results. The results of this study are limited to the ICUs in public training hospitals and it should be noted that there might be differences between the organizational culture as well as the supply of resources in public and private hospitals. Thus, it is essential to investigate the culture of the ICU nurses in private hospitals and compare their results with the findings of the present study.

## 5. Conclusion

The results indicated that in the context of the study, the ICU nurses are in a situation of uncertainty and supply shortage. Maintaining the quality of patient care with little attention to hospital costs was the main focus of beliefs formed up in the ICU workplace regarding the health economics which had an impact on the performance of nurses. Nurses have made the commitment to provide an appropriate and high-quality care through storing the resources to ensure their availability, implementing a high volume of clinical guidelines, and making efforts to reduce patient costs. Therefore, it is necessary to improve the supply chain in the hospitals by the effective management of the resources and to make the supplies optimally available to ICU nurses when required. It also seems appropriate that the hospital’s strategic plan is revised according to cost-effectiveness perspective; so that it improves the cost of care services while maintaining the quality of care and patient safety. In this regard, it is important for ICU nurses to become familiar with the principles of heath care economics and productivity by applying an effective cost management program. NMs and clinical nurses must be involved in decision-making about unit cost management. However, considering motivational factors, for instance, Nurses may have a more effective role in cost-effectiveness of health care services.
